# Accuracy of Community Informant Led Detection of Maternal Depression in Rural Pakistan

**DOI:** 10.3390/ijerph18031075

**Published:** 2021-01-26

**Authors:** Shamaila Mohsin, Ahmed Waqas, Najia Atif, Muhamamd Waqas Rabbani, Shahzad Ali Khan, Samina Bilal, Maria Sharif, Amina Bibi, Siham Sikander

**Affiliations:** 1 Department of Community Medicine, Army Medical College, University of Medical Sciences, Punjab 46000, Pakistan; 2 Department of Public Health, Health Services Academy, Opposite National Institute of Health, Islamabad 44000, Pakistan; shahzad@hsa.edu.pk; 3 Institute of Population Health, University of Liverpool, Liverpool L69 3BX, UK; ahmed.waqas@liverpool.ac.uk; 4 Human Development Research Foundation, Punjab 46000, Pakistan; najia.atif@hdrfoundation.org (N.A.); samina.bilal@hdrfoundation.org (S.B.); maria.sharif@hdrfoundation.org (M.S.); amina.bibi@hdrfoundation.org (A.B.); 5 Department of Behavioral Sciences, Shifa College of Medicine, Tameer-e-Millat University, Islamabad 44000, Pakistan; waqasrabbani.scm@stmu.edu.pk

**Keywords:** accuracy, community, informant, detection, maternal, depression, Pakistan

## Abstract

Maternal depression is a global mental health and a public health priority. Despite the priority its active detection is still a challenge. We tested the accuracy of an adapted version of Community Informant Detection Tool for Maternal Depression (CIDT-MD) in rural settings of Pakistan. Using a single stage design, trained community informants (lady health workers and lay peers) identified women (pregnant and/or with children) with symptoms of probable depression using CIDT-MD. This was immediately followed by diagnostic interviews of all the women using the Structured Clinical Interview for the Diagnostic and Statistical Manual (SCID-V) for current major depressive episode by trained assessors, blinded to the outcome of CIDT-MD. Data were analyzed using Statistical Package for Social Sciences (Version 25.0, IBM Corp., Armonk, NY, USA) and FACTOR software (Version. 10.3.01, Virgili University, Tarragona, Spain). Descriptive statistics, factor analysis, validity, reliability and known group validity was conducted to evaluate the psychometric properties of the adapted CIDT-MD. In all, 425 women, with mean age of 28 years (SD = 4.7), participated. Nearly 10% were illiterate, while the rest (90%) had an education ranging from eight to 15 years of schooling. The majority (73.2%) of the participants had 1–3 children while only 17.4% had >3 children. The sensitivity and specificity of CIDT-MD in detecting depressive symptoms was 97.5% (95% CI: 94.2–99.1) and 82.4% (95% CI: 77.8–86.4) respectively. It’s positive predictive value (PPV), 77.3% (95% CI: 72.9–81.2) and the negative predictive value (NPV) was 98.17% (95% CI: 95.7–99.2). While factor analysis revealed high inter-item correlation for most items (0.62–0.77) with an adequately fair Kaiser-Meyer-Olkin (KMO) sampling adequacy (0.73), significant Bartlett’s test of sphericity (*p* < 0.001). Uni-dimensionality for the CIDT-MD based on one-dimensional congruence (0.97), explained common variance (0.85), excellent internal consistency (0.90), good criterion validity (Area Under Curve = 81%), tester-test reliability (0.87–0.89) and statistically significant known group analysis (*p* < 0.001). The adapted version of the Community Informant Detection Tool for Maternal Depression is a valid and a reliable tool for active case detection of maternal depression in rural settings of Pakistan.

## 1. Introduction

Maternal depression, due to its burden in low–middle income settings [1], is a public health priority for both mothers and children [2,3]. Despite the burden, it still remains underrecognized and faces a huge treatment gap [4]. However task shifting or task sharing is now an evidenced approach to bridge the treatment gap and deliver psychosocial interventions through lay health workers or lay peers [5,6,7]. The World Health Organization’s (WHO’s) mental health Gap Action Programme (mhGAP), reiterates that, in resource constrained settings, primary health care structures should be utilized for both detection and provision of mental health services as part of the task shifting agenda [8]. Research in low resource settings indicate that this raises an issue of women being missed in these primary care facilities as they have poorer health seeking behaviors and seldom access the facility to have themselves screened for depression [9,10].

A more proactive, community based, active case detection approach is needed. Such an approach of active case detection for common mental disorders exists and was developed by Jordon et al. in Nepal [11]. This community-based approach employs context-specific pictorial illustrations of key symptoms of common mental health problems linked with non-stigmatizing idioms and matching vignettes (aided with visual illustrations and two questions) to detect mental health problems like alcohol-use disorder, epilepsy, and psychosis) [11]. This approach was based on the premise that detection is best achieved by community informants through illustrative recognition and broadly matching the people the informants encounter in daily routine in their area of work and responsibility. The subsequent accuracy testing of the tool also revealed good internal consistency (α = 0.83) and reliability of the above mentioned conditions among the community [12].

Traditionally, maternal depression either involves its screening through valid and standardized tools such as Edinburgh Postnatal Depression Scale (EPDS) or the Center for Epidemiologic Studies Depression Scale (CES-D) or self-administered tools such as the Patient Health Questionnaire (PHQ-9) [13,14]. Although the use of these tools is simple and cost effective [14], certain barriers such as low literacy populations and administration time limit their use low resource settings [15,16]. More over majority of the health workers have neither the time nor training to administer such question based screening tool [17]. However, health workers called lady health workers (LHWs) in Pakistan are residents of the same community they serve and provide promotive and preventive maternal and child health services at the household level [18]. The lady health workers visit each household within their catchment area on a monthly basis (some households are visited more frequently depending on the situation and need). By virtue of the work and knowledge of households especially women, the LHWs are best placed for active case detection of maternal depression and subsequent link up of these women with the primary care facility medical staff for confirmation [6]. Keeping in line with the approach used in Nepal, a community informant based detection tool for maternal depression was adapted in Pakistan. Details of the adaptation process can be found elsewhere [19].

Detection tools that are used, need to have high degrees of accuracy when compared to a gold standard [20]. Psychometric properties such as sensitivity and specificity are common measures used to measure the accuracy of detection tools [21].The community informant detection tool developed in Nepal when compared with a diagnostic gold standard that is, the Composite International Diagnostic Interview (CIDI) was able to accurately assign case-ness for the majority of persons (64%) that matched with pictorial vignettes [12,22]. The goal of this active detection approach that was adapted in Pakistan [19], is not to make a specific diagnosis when used by Lady Health Workers of lay Peers but rather to identify mothers with maternal depression symptoms, with high degree of accuracy, so that they can seek timely advice and confirmation of diagnosis by the primary care physicians. Keeping in line with same strategy as used in Nepal we adapted the tool for active case detection for maternal depression in Pakistan [19].The CIDT-MD adaptation procedure has several steps. First, perceived causes and idioms of symptoms of depression, as well as physical and psychological effects were found out by in-depth interviews with depressed and recovered mothers, mothers-in-laws and community health workers (lady health workers and lay peers). Subsequently, an inventory of local idioms and perceived causes was made, and selection of most relevant idioms was done through prioritization by an expert panel of Pakistani mental health professionals. Based on the analysis; vignettes for symptoms of depression and perceived causes was made and to facilitate the process of prompt recognition of people that potentially match the vignette, pictorial illustrations were developed for individual symptoms of maternal depression. In order to assess the comprehensibility of the tool focus group discussions was done with the end users community health workers (lady health workers and lay Peers) subsequently the tool was finalized by an expert panel of Pakistani mental health professionals [19]. The current paper focuses on reporting the accuracy of the Community Informant Maternal Depression Detection Tool (CIDT-MD) used by community-based informants that is Lady Health Workers and lay Peers in in the community settings of Pakistan. However, despite its nature as an active detection tool, CIDT-MD is essentially a tool to assess probable depression. Moreover, according to traditional psychometrics, the tool has data pertaining to a set of symptoms (observed variables) for the detection of depression (latent construct). Hence, it was important that all relevant steps be taken to ensure that the instrument is valid and reliable. Therefore, we present analyses to establish different facets of accuracy (validity and reliability) in this manuscript.

## 2. Material and Methods

### 2.1. Setting

The study was conducted in one of the seven rural sub-districts of the District Rawalpindi, called Kallar Syedan in Punjab, Pakistan. The sub-district has 11 Union Councils (UCs). A Union Council is the smallest administrative unit consisting of 15–20 villages and has a population of about 22,000–25,000. Each UC is serviced by a primary health care facility called the Basic Health Unit (BHU). There are 122 lady health workers (LHWs) in the Kallar Syedan sub-district, covering approximately 95% of the population. The average household, within the sub-district, consists of 6.2 members. The sub-district is representative of a typical low-socioeconomic rural area of Pakistan. This area was selected for a number of reasons as it is geographically, culturally, and socio-economically similar to many other sub-districts of the province and country. It is one of the first districts of Pakistan where the WHO mhGAP has been rolled-out as a pilot and a maternal depression birth cohort and a randomized trial trained peers delivering a perinatal depression psychosocial intervention were ongoing [23,24]. Leveraging all of these on-going activities and studies, one third of UCs (3 out of 11) of Kallar Syedan were purposively selected for the adaptation and accuracy testing of CIDT-MD.

### 2.2. Recruitment of LHWs and Peers

In the first instance, the 44 LHWs and 18 peers (*n* = 62) within the selected three UCs were approached to take part in the study. We ensured they fulfilled the criteria of being a community informant which meant: (a) they had to have an in-person contact with the mothers within the last one month and be aware of their client’s overall health condition and (b) be knowledgeable of their overall socio-economic and household circumstances.

### 2.3. Instruments

#### 2.3.1. CIDT-MD

The Community Informant Maternal Depression Tool (CIDT-MD) was adapted after a multistep iterative process. Details of the adaptation process are reported elsewhere [19]. The tool has three sections. The first section has a set of nine illustrations based on the women/mothers experiencing symptoms of depression. These symptoms being (i) difficulty in sleeping, (ii) loss of appetite, (iii) agitation, (iv) lack of concentration, (v) helplessness, (vi) fatigue, (vii) loss of interest, (viii) low mood and (ix) suicidal ideation. All done in a culturally appropriate and sensitize manner. The second section of CIDT-MD has a set of visual analogue based Likert scale questions for the informants to rate. The first question is about the likelihood of matching of illustrated symptoms on the tool vs. the symptoms the informant knows about. The 4-point Likert scale being (i) no match, (ii) some match, (iii) most match and (iv) complete match. Then there were three more questions with binary (Yes/No) response pertaining to daily functioning, chronicity, or recurrence and suicidal ideation. The third section consists of need for help seeking in case of depression. The mother and family are informed by the community informant regarding the set of symptoms and the need to seek further help from the primary care physician.

#### 2.3.2. Structured Clinical Interviews of DSM Disorders (SCID)

The SCID module for current major depressive episode was used as the gold standard to test the accuracy of CIDT-MD. The SCID is a semi-structured interview that generates case vs. non-case diagnosis of current major depressive episode by inquiring about the individual symptoms of depression based on the Diagnostic and Statistical Manual (DSM) classification of disorders. To make a diagnosis of depression at least five symptoms are needed, including depressed mood or loss of interest [25]. The SCID module of depression within the perinatal populations has been used extensively adapted and across culturally validated and can be used by trained non-mental health specialists and has been used extensively in the study area site [26,27,28,29].

#### 2.3.3. Training of Community Informants to Use CIDT-MD

**Figure 1:**
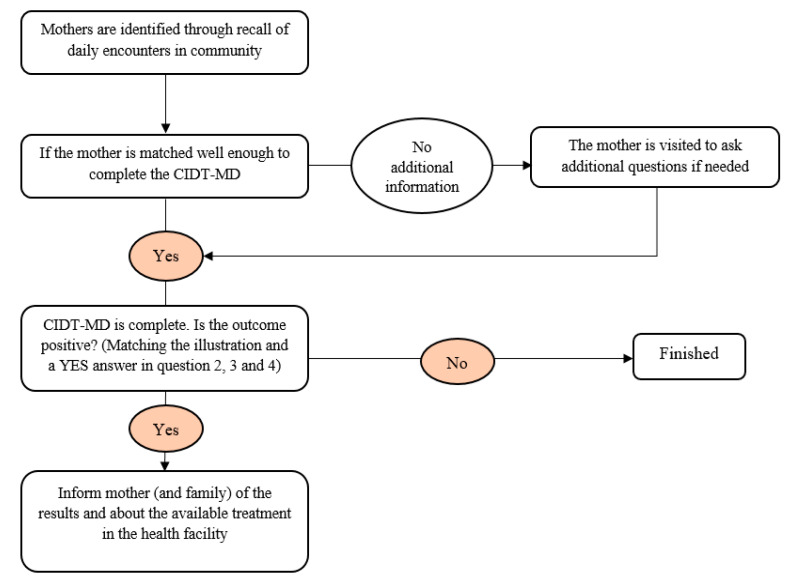
Summary of Detection of Mothers by Community Informants. CIDT-MD is Community Informant Detection Tool for Maternal Depression.

Based on this criteria the selected Community Informants (i.e., LHWs and Peers) were given training in a two hour session on the Community Informant Detection Tool for Maternal Depression (CIDT-MD) at the Basic Health Units of the three UCs in sub-district Kallar Syedan. The training covered introduction to maternal depression, brief discussion of the symptoms of depression and stigma associated with mental health problems. Followed by role plays to enhance learning and coding to the tool. The community informants were trained to use the tool in four steps: identification, matching, day to day functioning/self-harm, and help seeking. This classroom-based training was followed by field practice sessions. Each community informant were asked to fill out the tool for at least four women within their respective catchment areas. This helped to address issues and ambiguities in items and scoring rubric of CIDT-MD as shown in Figure 1.

### 2.4. Data Collection Procedure

**Figure 2:**
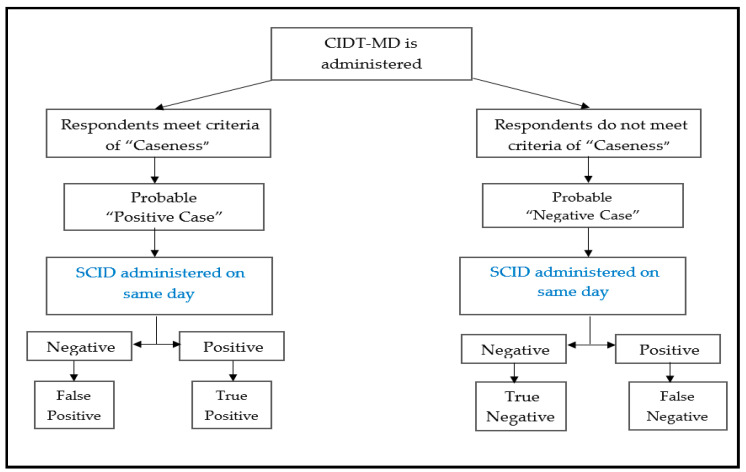
The Schematic Illustration of Assigning Caseness. (SCID is Structured Clinical Interviews of DSM Disorders).

After the training and field practice, data collection took place over 4 months (June–September 2019). Using a single stage design in which all 62 community informants (LHWs and Peers) carried out active case detection of “probable cases” or “probable negative cases” in their respective fields among pregnant women and mothers with up to three years olds. There were no refusals among the community informants and since they interact with the mothers frequently at the household level so they fulfilled the criteria of recruitment. All the set of trained community informants (which included both LHWs and Peers) were given a list of women eligible to have their individual CIDT-MD tools filled out on a given day. This was done separately as this did not require any interaction between the informants and the women on the list. Simultaneously, all these women on the list, on the same day, were assessed by trained female assessors at household for depression, using the SCID diagnostic interview. These women were only interviewed for their diagnosis by the assessment team. Community informants just filled out the tool based on their knowledge of the women’s symptoms. This approach had minimalistic respondent fatigue which contributed to a high response rate. See Figure 2.All data were collected electronically.

### 2.5. Sample Size

Based upon the literature review, the sample size was calculated using the formula:

*n_Se_* = *z*^2^ × *Se* (1 − *Se*)/*d*^2^ × *Prev* (1)

The sensitivity (*Se*) of the tool used in Nepal was taken as reference 92%, with a 95% confidence interval (*z* = 1.96) and the population prevalence (*Prev*) of Maternal depression as 26% and an accuracy (*d*) of 0.05 the sample size calculated was 435. The sample size was also calculated with the specificity (*Sp*) of the tool used in Nepal taken as a reference, that is 66% with a 95% confidence interval (*z* = 1.96), and the population prevalence of maternal depression as 26% and a precision (*d*) of 0.05 using the formula:

*n_Sp_* = *z*^2^ × *Sp* (1 − *Sp*)/*d*^2^ × (1 − *Prev*) (2)

The sample size was 466. To find the accuracy of the tool the value of 435 was taken as the sample size.

### 2.6. Ethics

Ethical approval was taken from institutional review board (IRB) of Human Development Research Foundation (HDRF) and Health Services Academy (HSA) (Ref No IRB/004/2019).

### 2.7. Study Variables

The study variables introduced in the data analyses included individual items on the CIDT-MD tool, diagnosis of depression according to SCID current major depressive episode and social stressors: which included being overwhelmed with domestic responsibility, poverty, ailments, and experience of domestic violence.

### 2.8. Data Analysis

All data were analyzed using SPSS (Version 25.0, IBM Corp., Armonk, NY, USA) and FACTOR software (Version. 10.3.01, Virgili University, Tarragona, Spain). For all the tests a maximum error of 5% was accepted.

#### 2.8.1. Descriptive Statistics

Socio-demographic, maternal characteristics were given as frequencies (percentages) and mean (S.D). The participants identified with depressive symptoms by Community Informants were compared with those without depressive symptoms verified by SCID across various socio-demographic characteristics using the chi square test, in the case of categorical data.

#### 2.8.2. Face Validity of CIDT-MD

It was done by an expert panel consisting of consultants in psychiatrists, psychologists and public mental health experts. They convened the final adaptation process. In addition an Item impact measurement technique was also used. The panel of experts scored the importance of each item with a 5-point Likert scale, from slightly important (score1) to very important (score 5).

#### 2.8.3. Content Validity

Quantitative content validity was evaluated by the content validity ratio (CVR) and content validity index (CVI). For CVR calculation, experts assessed the item essentiality. The score of each item of the Community Informant tool was considered within a three-degree range of “not essential, useful but not essential, essential” from 1 to 3 points. CVR varies between 1 and −1. The total score of CVR was determined by Lawshe table (1975) and based on the number of the expert. In this study, 5 experts were included, so any item with a CVR of more than 0.5 was accepted.

#### 2.8.4. Construct Validity

Principal factor analysis (PFA) was used to evaluate the construct validity with polychoric correlations to evaluate the latent constructs of CIDT-MD. In order to evaluate the adequacy of sampling to perform exploratory factor analysis, sample size was important, so KMO test and Bartlett’s sphericity test was used to confirm the adequacy of sampling in PFA. The KMO index ranges from 0 to 1. KMO more than 0.7 is interpreted as acceptable and large sample size that was suitable for EFA. The Bartlett’s test of sphericity should have significant results (*p* < 0.05).

The suitability of each item for inclusion in the factor analyses was judged using several criteria: KMO measure for individual items >0.5 in anti-image matrix and polychoric correlation of at least 0.2 for individual items, revealed by the covariance matrix. Thereafter, number of factors to retain were based on three criteria: Eigen value >1, proportion of variance explained by each factor, visualization of Cattel’s scree plot and Horn’s parallel analysis. Suitability of each item for inclusion in the scale, was assessed using the communality value >0.20 and a minimum factor loading of 0.32.

#### 2.8.5. Goodness of Fit

Goodness of fit was assessed using the root mean square of residuals (RMSR) and weighted RMSR. It was decided that, if the value of RMSR is much larger than Kelley’s criterion value, the model would not be considered as good. While for weighted RMSR, values under 1.0 have been recommended to represent good fit.

#### 2.8.6. Reliability

Internal consistency and stability was used to verify the reliability of CIDT-MD. Internal consistency would be estimated by computing Cronbach’s alpha coefficient for CIDT-MD. The alpha values of 0.70 or above was considered as acceptable. Item-scale correlations corrected for overlaps using Pearson’s product moment correlation coefficient, were considered acceptable if ≥0.2. In addition, the McDonalds Omega score was also assessed. Test-retest reliability of CIDT-MD and its subscale for two-week interval was estimated by intra-class correlation coefficient (ICC). ICC values of 0.7–0.8 was considered as having suitable stability.

#### 2.8.7. Criterion Validity

The criterion validity of the CIDT-MD as assessed using SCID (gold standard) comparator tool. For this purpose, the receiver operating curve analysis was run, where an area under curve of 0.80 was considered as having an excellent discrimination capacity of the scale. Suitable cut-off point and corresponding sensitivity and specificity for the scale was explored at this stage.

The results from the CIDT-MD and the SCID was compared and plotted as true or false positives and true or false negatives. To verify the association between CIDT-MD and SCID, we assessed the criterion-related accuracy of CIDT-MD to SCID by calculating sensitivity, specificity, positive predictive value (PPV), and negative predictive value (NPV).

#### 2.8.8. Known Group Validity

It is the ability of an instrument to be sensitive to the differences between groups of participants that may anticipate the score differently in the predicted direction. This was conducted for the perceived social causes of depression (overwhelmed with domestic responsibility, poverty, physical ailments, and experience of domestic violence).

## 3. Results

### 3.1. Participant Characteristics

A total of 435 women were approached, for detection for maternal depression. In all there were 10 refusals out of the 435 (*n* = 425). The mean age of the women was 28 years (SD = 4.7). Nearly 10% were illiterate the rest had education ranging from 8–10 years of schooling or above. The majority (73.2%) of the participants had 1–3 children while only 17.4% had more than three children. Table 1 shows the association of various demographic characteristics of depression measured by the Community Informant Detection Tool for Maternal Depression (CIDT-MD). Using the CIDT-MD, the probable positive (*n* = 150) and/or probable negative cases (*n* = 275) were identified by the informants. After SCID, was administered, 95 (63.3%) of the CIDT-MD positive cases (true positives) and 268 (98.1%) of the CIDT-MD negative cases (true negatives) were found to have meet the diagnostic criteria for current major depressive episode on SCID. 

### 3.2. Face Validity

We used a multistep qualitative iterative approach to culturally adapt the Community Informant Detection Tool for Maternal Depression for administration by lay health workers (process and scoring found elsewhere [19]. The process entailed five evidence informed steps, namely the selection of an appropriate tool for adaptation through scoping review of literature, detailed formative research to explore the perceptions of key informants, formulation of culturally appropriate illustrations, feedback of end users on the barriers and facilitators of detection, and finally approval of the detection tool by panel of mental health experts. The panel of experts in psychiatry and public health convened the final adaptation process by rephrasing the items and making each response more comprehensible for lay health care workers. Four items were reworded based on the general consensus of the panel. Of particular importance was the rewording of items to reflect the specificity of the symptoms of maternal depression. Each expert reflected the distinct insight into the tool adaptation process (blind to the other members’ feedback) provided detailed comments on suitability of the detection tool to be used by the community informants at each step. The qualitative responses from the panel indicated that the tool appeared to detect maternal depression and was psychometrically feasible, indicating adequate face validity.

### 3.3. Content Validity

The mean CVR across all items was 0.76 which is indicative of good content validity. Two items from the symptom illustration was deleted either due to repetition or because there was general agreement that they did not specifically relate to maternal depression (e.g., low CVR). In addition, the graphic Likert scale (water glass analogy) was included that was to aid the limited understanding of the community informants perceived by the panel. The design and presentation of the final tool was then extensively reviewed to ensure it was streamlined and easy to respond to.

### 3.4. Construct Validity

Factor validation analyses was conducted in two phases. We conducted principal component analysis with poly-choric correlations for the CMIDT scale comprising of items pertaining to symptoms of depression, chronicity, recurrence and day to day functioning. In present analyses, the KMO sampling adequacy measure was found to be fair (0.73) and Bartlett’s test of sphericity was statistically significant (*p* < 0.001). Assessment of the covariance matrix (poly-choric correlations) revealed that all items has at least one inter-item correlation of 0.2. Assessment of Eigen values revealed that two factors had an Eigen value >1; the first factor had an Eigen value of 5.90 that explained 49.20% of the variance in the CMIDT. While the second factor yielded an Eigen value of 1.18 explaining only 9.8% variance, Horn’s parallel analysis based on Principal Component Analysis suggested that only one factor be retained. All of the items yielded communality values >0.2 and factor loading >0.32.

No floor and ceiling effects were noticed in CMIDT assessment measure for depression. A total of 66 (15.5%) of the participants scored the minimum score of 0 and 9 (2.1%) the highest j. If a significant proportion of people have scores at the bottom (floor) or top (ceiling) of the range of possible scores, then the potential responsiveness of tool will be impaired as it will not necessarily measure change.

### 3.5. Uni-Dimensionality Measures & Goodness of Fit

The overall assessment of uni-dimensionality for the CMIDT was based on the values of one-dimensional congruence (0.97), explained common variance (0.85) and mean of item residual absolute loadings 0.25. All these statistics suggested that the data should be treated as uni-dimensional. Goodness of fit was assessed using RMSR by taking into account Kelley’s criterion and weighted RMSR considered acceptable at 1.0.

### 3.6. Reliability

The Internal consistency between CIDT-MD items was excellent. The standardized Cronbach’s revealed excellent internal consistency valued for all 18 items was 0.90. The Cronbach’s alpha co-efficient for CIDT-MD domains were as follows: Illustrations of symptoms of depression (nine items), 0.92; matching to Pictorial illustrations (five items), 0.87 respectively. This was also revealed by excellent values of McDonald’s ordinal Omega (0.90) and greatest lower bound to reliability (0.97). However, the GLB and Omega are more appropriate in large samples due to positive sampling bias. The inter-data collector reliability was assessed by correlating the results of two observations for the same mother by two lay health care workers. The ICCs for the inter-data collector reliability of CIDT-MD ranged from 0.87 to 0.89 (*p* < 0.01). (Table 2). Reliability analyses also identified several items with poor alpha coefficient, which were excluded from the factor analyses. These items pertained to the social stressors and recurrence of depressive symptoms.

### 3.7. Criterion Validity

**Figure 3:**
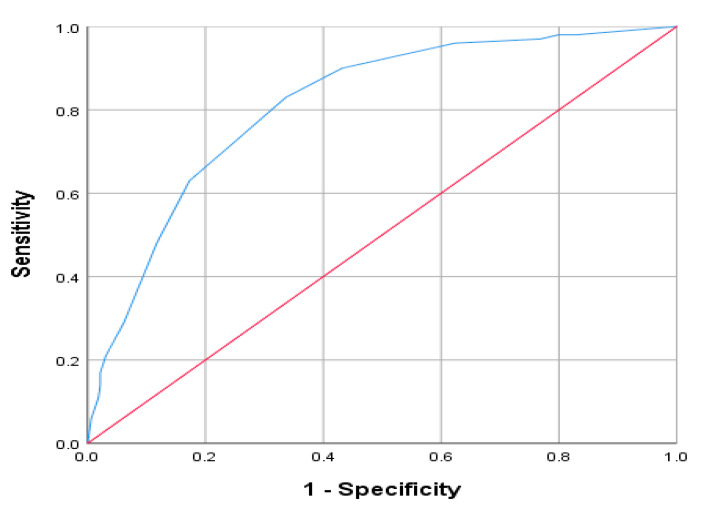
Receiver Operator Curve (ROC)-presenting the relationship between sensitivity and specificity of CIDT-MD for every possible cutoff. (Blue curve = ROC curve, Red Line = chance line).

Received operating curve analysis (Figure 3) comparing CIDT-MD scores with gold standard comparator of SCID based on the sum scores of CIDT-MD reveled an excellent value for area under curve of 81.50%. A cut off value of 3.5 on CIDT-MD was revealed as having a good sensitivity value of 81% and specificity of 69.4%.

Whereas, the criterion related scores based on complex scoring criteria where the presence of one symptom was rated as a probable case of depression revealed the sensitivity of CIDT-MD to be 97.5% (95% CI: 94.2–99.1) and specificity was 82.4% (95% CI: 77.8–86.4). Similarly the positive predictive value (PPV) was 77.3% (95% CI: 72.9–81.2) and the negative predictive value (NPV) was 98.17% (95% CI: 95.7–99.2). 

### 3.8. Known Group Validity

In known group validity, an independent sample t test revealed that women who experienced poverty, physical ailments, domestic experience violence, and felt overwhelmed with domestic responsibility scored significantly higher on CMIDT. There was a significant difference between both groups in terms of perceived social causes (*p* < 0.05) as shown in Table 3.

## 4. Discussion

This study provides the evidence that the Community Informant Detection Tool for Maternal Depression (CIDT-MD) satisfies the criteria for an active or pro-active case detection tool for an important public health priority condition through community workers. This paper shows that accurately assigning case-ness among depressed pregnant women and mothers through a pictorial vignettes based approach works well. CIDT-MD exhibited good psychometric properties, including high reliability and exhibited adequate sensitivity when compared with a diagnostic gold standard. It has a simple unidimensional factor structure and showed good content and face validity. This pro-active case detection tool is therefore culturally appropriate and valid for community settings of Pakistan and is likely to be applicable in other similar settings.

The tool exhibited excellent sensitivity (0.97) and specificity (0.82) of detection for maternal depression among rural pregnant women and mothers with up to three year olds. The results suggest better accuracy compared to the Community Informant Tool for Detection of Depression in Nepal [12]. However, these findings are in contrast with some existing screening tools, such as Patient Health Questionnaire -9 (PHQ-9), Center for Epidemiological Studies-Depression,(EPDS), Center for Epidemiological Studies-Depression (CES-D), etc., that have exhibited higher sensitivity in certain studies [30,31,32,33,34,35,36,37,38,39]. However, there are concerns that in resource constrained settings in case of universal screening this higher sensitivity would come at the expense of high false-positive rates that would undermine the cost effectiveness of using screening tools. The positive and negative predictive values (PPV = 0.77, NPV = 0.98) of the CIDT-MD for detecting maternal depression varied slightly in comparison with CIDT in Nepal (PPV = 0.64, NPV 0.93) respectively [12]. Similarly the estimate of the positive likelihood ratios for the detection of depression in our sample was high (5.56) in comparison to CIDT (2.71) in Nepal. The degree of variation found is likely to be due to the prevalence rates of depression, the difference in diagnostic criteria and language of administration used across these studies. Perhaps other reasons could be that the Nepalese tool had more than one disorder pro-actively detected and using a two staged design to assess the accuracy of their tool [12].

The preliminary feedback from our set of community informants during the training and subsequent supervision of CIDT-MD, indicated that sufficient information was given in a relatively shorter duration, that was found very useful (thus acceptable and feasible to use and adopt). CIDT-MD was also regarded simple in its use since clear pictorial representations of many of the symptoms and vignettes made it easy to understand by the community informants [19]. This puts it at an advantage of doing relatively longer training sessions, extensive supervision, and cost required for the administration of screening tools at a mass scale [15,40,41].It was conjunction to the evidence that suggests that the approaches that incorporated the use of community informants (LHWs, peers etc.) have exhibited the potential and capacity to identify maternal depression [42]. This is not to mention that such a pro-active case detection approach also holds population level advantages due to greater population being covered at a household level by community-workers, especially in low-income settings like Pakistan.

In comparison to the existing screening tools that are based on the DSM-IV diagnostic criteria and ICD-10 criteria [15,39] this proactive case detection is based on frequency of symptom detection [43]. This simplified tool with prototype matching with dichotomous response on individual symptoms has demonstrated comparable validity to screening tools based on scoring criteria and cutoff points. In practice, such active case-finding enhances the accessibility of mental health services in settings, such as Nigeria and India. [44,45].

A priori sample size estimation was done for the current study which reflects the findings of a recent systematic review in which only less than 10% of validation studies used this recommended method for sample size determination to achieve given precision [46]. Secondly, using a single stage design in which same day diagnostic interviews were done by masked assessment team for all the participants as opposed to just the “high scorers” A single stage design helps address possibility of measuring different symptom profiles in the same individual, given the potential for fluctuations in the mental state within a period of 24 h [47].

The factor structure of the adapted CIDT-MD was rigorously evaluated and found to explicitly exhibit unidimensional measure of depression indicating a good homogeneity of items. The evaluation of the factorial structure is the first step in the accuracy study [48]. Conversely, some other studies analyzing screening tools yielded multi-factorial construct [49,50]. Differences may be explained by the fact that the screening tools are mostly tested in population with different ethnicities and cultures, and analysed with diverse rotation methods and factor loading coefficients. The construct validity of the CIDT-MD was analysed using the polychoric factor analytic technique that had a favorable internal consistency; as evidence indicates that polychoric correlation is an ideal analytic technique for variables that are dichotomous or ordinal [51]. Conversely, evidence indicates that a number of studies used principal component analysis, a variable reduction technique that may not be appropriate for underlying factor structure of psychometric instruments [52]. The results are remarkably similar to the other studies conducted using the Community Informant Tool for Detection (CIDT) in similar low resource settings in Nepal and Kenya [12,53]. The psychosocial stressors identified among the participants in the adaptation study were inter-personnel conflicts, domestic abuse, poverty, and physical ailments. Literature also confirms that in LMIC there is relatively high prevalence of maternal depression due to exposure of women to multiple stressors such as poverty, lack of social support, violence, conflict, migration, disasters, and exposure to intimate partner violence

### 4.1. Future Implications

While the findings of this study need to be replicated in other settings, the study has several implications for the field of global mental health specially to help pro-actively detect maternal depression. The strong sensitivity and specificity of the tool further strengthens the argument that active case detection for maternal depression can be done feasibly and reliably through community informants. The CIDT-MD is easy to administer in settings such as Pakistan where mental health systems are non-existent. Pakistan has nearly a hundred though Lady Health workers (90,000–110,000) [54] covering nearly 80% of the rural population can be a source for active case detection for maternal depression among women and mothers to help link up with health systems and appropriate management. However, in their current workload, they can effectively administer active cased detection tools that can be integrated into their routine work. As it has been proven effective, CIDT-MD may not only be used in similar areas in Pakistan, but rolled out to other areas for further adaptation. CIDT-MD may be adapted in different formats (e.g., mobile app-delivered) and different local languages (Sindhi, Balochi, Pashto) across a variety of populations afflicted with maternal depression.

### 4.2. Strengths & Limitations

This study has several strengths including use of a single stage design with a diagnostic interview as the gold standard, masked assessments, culturally adapted tool, appropriate sample size, broader inclusion criterion (perinatal women and women with 3 year old children), including both cases and non-cases (participating in the diagnostic interview stage) and finally using robust analytical approaches to illustrate accuracy including identifying the factor structure of the CIDT-MD and its goodness of fit, several facets of validity including face validity, content validation, and criterion validity. However, these results need to be interpreted with caution as the generalizability of this community informant detection in the absence of existing community-based workers (e.g., LHWs and/or Peers) might be poor because LHWs work in close collaboration with the community and knowledgeable of issues and conditions of the communities they serve. Similarly, the generalizability of the findings to populations within Pakistan that do not speak Urdu will also be limited.

## 5. Conclusions

This research shows that CIDT-MD is an accurate and a feasible tool to use for pro-active case detection of maternal depression in low resource settings using community informants. The CIDT-MD is easy to administer in settings such as Pakistan where mental health systems are non-existent. Pakistan has approximately 100,000 lady health workers covering nearly 80% of the rural population. The LHWs can become a huge resource to help active case detection for maternal depression and help link them up with health systems and eventually reduce the treatment gap of maternal depression.

## Figures and Tables

**Table 1:** Demographic characteristics of study participants (*n* = 425).

Variables	CIDT-MD		*p*-Value	SCID		*p*-Value
	Depressed	Non Depressed		Depressed	Non Depressed	
	*n* = 150 (35%)	*n* = 275 (65%)		*n* = 100 (23.5%)	*n* = 325 (76.4%)	
Women’s Age						
18–27	65 (43.3%)	128 (46.5%)	0.07	46 (46%)	147 (45.2%)	0.84
28–37	79 (52.6%)	139 (50.5%)		52 (52%)	168 (51.7%)	
≥38	6 (4%)	8 (3%)		2 (2%)	10 (3.1%)	
Family Structure						
Joint	65 (43.3%)	107 (39%)	0.05	47 (47%)	157 (48.3%)	0.63
Multiple Households	26 (17.3%)	30 (11%)		14 (14%)	125 (38.4%)	
Nuclear	58 (30.6%)	136 (49.4%)		38 (38%)	43 (13.2%)	
Number of Children						
None	8 (5.3%)	33 (12.1%)	0.06	8 (8%)	33 (10.2%)	0.23
1–3	107 (71.3%)	207 (75.2%)		58 (58%)	243 (74.7%)	
>3	35 (23.3%)	35 (12.7%)		26 (26%)	49 (15%)	
Women Education						
None	20 (13.3%)	21 (7.7%)	0.02	13 (13%)	26 (8%)	0.004
Primary	28 (18.7%)	31 (11.4%		20 (20%)	38 (11.6%)	
Middle	23 (15.3%)	29 (10.6%)		15 (15%)	40.2(13%)	
Secondary	40 (26.7%)	95 (34%)		27 (27%)	103 (31.6%)	
Higher Secondary	23 (15.3%)	34 (12.5%)		15 (15%)	43 (13.2%)	
Graduate	12 (8%)	44 (16.1%)		9 (9%)	48 (15%)	
Masters	4 (2.7%)	22 (8.1%)		1 (1%)	25 (8%)	
Husband Education						
None	16 (10.7%)	12 (4.4%)	0.05	10 (10%)	18 (5.5%)	0.04
Primary	13 (8.7%)	19 (7%)		11 (11%)	21 (6.5%)	
Middle	24 (16%)	53 (19.4%)		21 (21%)	56 (17.2%)	
Secondary	75 (50%)	119 (43.1%)		42 (40.2%)	152 (46.2%)	
Higher Secondary	13 (8.7%)	40 (14.7%)		9 (9%)	44 (13.5%)	
Graduate	6 (4%)	20 (7.3%)		4 (4%)	22 (6.8%)	
Masters	3 (2%)	11 (4%)		3 (3%)	12 (3.7%)	
Income						
Did not know	21 (14%)	36 (13.1%)	0.23	13 (13%)	45 (13.8%)	0.36
10000–20,000	46 (30.6%)	114 (41.4%)		32 (32%)	128 (45.5%)	
21,000–30,000	49 (12.6%)	78 (28.3%)		35 (35%)	92 (28.3%)	
31,000–40,000	24 (16%)	30 (11%)		14 (14%)	41 (12.6%)	
41,000–50,000	7 (4.6%)	12 (4.3%)		3 (3%)	16 (5%)	
>51,000	3 (2%)	3 (1.1%)		3 (3%)	3 (1%)	

**Table 2:** Reliability and Factor Validity Statistics of Tool (After Deletion of Redundant Items).

Items	Corrected Item-Total Correlation	Squared Multiple Correlation	Cronbach’s Alpha if Item Deleted	Factor Loading	Communality
Difficulty in sleeping	0.783	0.655	0.853	0.64	0.42
Loss of appetite	0.750	0.626	0.854	0.57	0.32
Agitation	0.703	0.532	0.857	0.59	0.35
Lack of Concentration	0.778	0.663	0.853	0.67	0.45
Helplessness	0.730	0.605	0.856	0.57	0.32
Fatigue	0.689	0.551	0.858	0.68	0.46
Lack of Interest	0.581	0.413	0.866	0.76	0.58
Low mood	0.767	0.698	0.854	0.84	0.70
Suicidal ideation	0.374	0.495	0.873	0.85	0.71
Day to day functioning	0.157	0.309	0.877	0.80	0.67
Chronicity	0.312	0.322	0.873	0.65	0.42
Self-harm/Suicidal Ideation	0.113	0.274	0.879	0.73	0.54

**Table 3:** Known Group Validity showing Association between CIDT-MD and Perceived Cause (Psychosocial Stressors).

Stressors	Response	Mean	Standard Deviation	*p*-Value
Overwhelmed with domestic responsibility	No	3.67	3.19	<0.001
	Yes	4.70	2.65	
Poverty	No	3.82	2.96	<0.001
	Yes	5.60	2.64	
Ailments	No	3.83	3.06	<0.001
	Yes	5.13	2.55	
Experience of domestic violence	No	3.86	2.78	<0.001
	Yes	8.28	2.71	

## Data Availability

The data presented in this study are available on request from the corresponding author. The data are not publicly available due to privacy.
